# Shikonin: From Biosynthesis and Pharmacological Mechanisms to Translational Safety and Drug Delivery

**DOI:** 10.1002/cbic.70586

**Published:** 2026-11-05

**Authors:** Namini Joshi, Prabhakar Semwal, Balwant Rawat, Ravendra Kumar, Mozaniel Santana de Oliveira, Nishant Rai, Janhvi Mishra Rawat

**Affiliations:** ^1^ Plant Biotechnology Laboratory, Department of Biotechnology Graphic Era Deemed to be University Uttarakhand India; ^2^ School of Agriculture Graphic Era Hill University Dehradun Uttarakhand India; ^3^ Department of Chemistry College of Basic Sciences and Humanities G.B. Pant University of Agriculture and Technology U.S. Nagar Uttarakhand India; ^4^ Departamente de Química Instituto Federal de Educação Ciência e Tecnologia do Pará (IFPA) Redenção Brazil

**Keywords:** nanomedicine, naphthoquinone, regulated cell death, shikonin, translational pharmacology

## Abstract

Shikonin (SHK) is a red naphthoquinone produced mainly in the roots of Boraginaceae species and has been investigated for anticancer, anti‐inflammatory, antimicrobial, neuroprotective, cardioprotective, hepatoprotective, and wound‐healing activities. This review critically integrates botanical sources and biosynthesis with pharmacological mechanisms, preclinical evidence, safety, and delivery strategies. Across cancer models, SHK converges on redox imbalance and regulated cell‐death programmes, including apoptosis, cell‐cycle arrest, autophagy, necroptosis, ferroptosis, and immunogenic cell death, while modulating NF‐κB, MAPK, PI3K/Akt, and related signalling networks. However, the evidence is dominated by heterogeneous in vitro experiments and small‐animal studies, with limited pharmacokinetic standardisation, incomplete assessment of selectivity toward diseased versus normal tissues, and almost no robust clinical evaluation. Poor aqueous solubility, chemical and photochemical instability, rapid metabolism, route‐dependent exposure, and a context‐dependent therapeutic window remain central translational barriers. Liposomes, polymeric micelles, nanoparticles, nanogels, nanoemulsions, and ligand‐ or stimulus‐responsive systems can improve dispersion, tissue accumulation, controlled release, and combination therapy; nevertheless, their long‐term safety, manufacturing reproducibility, and comparative advantage over free SHK require rigorous validation. SHK should therefore be regarded as a mechanistically versatile therapeutic lead rather than a clinically established treatment. Translation will depend on exposure–response studies, standardised formulations, clinically relevant models, and well‐designed human trials.

## Graphical abstract

Shikonin, biosynthesised in Boraginaceae roots from phenylalanine‐derived PHB and isoprenoid GPP, modulates redox balance, cellular stress, signalling pathways, and regulated cell death. Preclinical studies support diverse pharmacological activities, while nanocarriers improve dispersion and delivery. Translation requires standardised materials, exposure‐guided safety assessment, reproducible manufacturing, and rigorous clinical validation before therapeutic application.

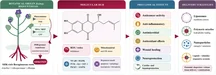

## 1 Introduction

Shikonin (SHK) is a red naphthoquinone found in the pigmented roots of several Boraginaceae species and in traditional topical preparations [1, 2]. Comparative chromatography has quantified multiple SHK‐type derivatives in different Boraginaceae herbs [3], while cellular pharmacology studies have shown that these derivatives do not necessarily produce the same biological responses [4]. These findings make chemical identity a central consideration: observations from an extract or an acylated derivative cannot automatically be attributed to purified SHK.

Several experimentally observed effects provide a basis for mechanistic hypotheses. In cancer‐cell models, SHK induced mitochondrial dysfunction, reactive oxygen species (ROS) production, and loss of membrane potential [5]; another study demonstrated inhibition of pyruvate kinase M2 and cancer‐cell glycolysis [6]. ROS‐dependent apoptosis has also been observed in primary effusion lymphoma cells [7], whereas proteasome inhibition and altered inflammatory signalling have been reported in macrophages [8]. Together, these results motivate the hypothesis that metabolic and redox responses to SHK depend on cellular context and exposure. Pathway‐marker changes alone, however, cannot establish direct target engagement in every model.

The range of investigated applications likewise requires separate evaluation of each model. An engineered SHK/chitosan‐silver nanocomplex induced necroptotic immunogenic cell death in a breast‐cancer model [9]; other studies examined antibacterial effects against methicillin‐resistant *Staphylococcus aureus* [10] and skin repair in mice [11]. These results support preclinical investigation, but they do not establish efficacy of the same purified formulation across indications. A historical human report evaluated a SHK mixture in lung cancer [12], so it cannot isolate the clinical effect of SHK itself.

Safety and formulation may further determine whether these preclinical effects are transferable. SHK and acetylshikonin showed different cytotoxicity toward a normal‐cell line [13]. The photoreaction of SHK has been investigated in solution and in the solid state [14], and surfactant‐coated nanoparticles improved its stability against heat and light and its in vitro availability [15]. SHK/anthracycline liposomes have also been tested for immunogenic cell death in an experimental tumour model [16]. This evidence supports a second working hypothesis: any therapeutic benefit will depend on defined formulation, adequate exposure at the target tissue, and an acceptable safety margin, each of which requires direct measurement.

This review connects botanical sources and biosynthesis to the pharmacological evidence for SHK, then examines preclinical models, the limited human evidence, safety, and drug‐delivery approaches. Throughout, it distinguishes purified SHK from extracts and derivatives, evaluates the strength of mechanistic claims, and identifies the exposure, selectivity, and formulation questions that remain before clinical development. The accompanying frontispiece provides a visual overview of SHK's botanical origin, molecular structure, and the preclinical activities examined in this review.


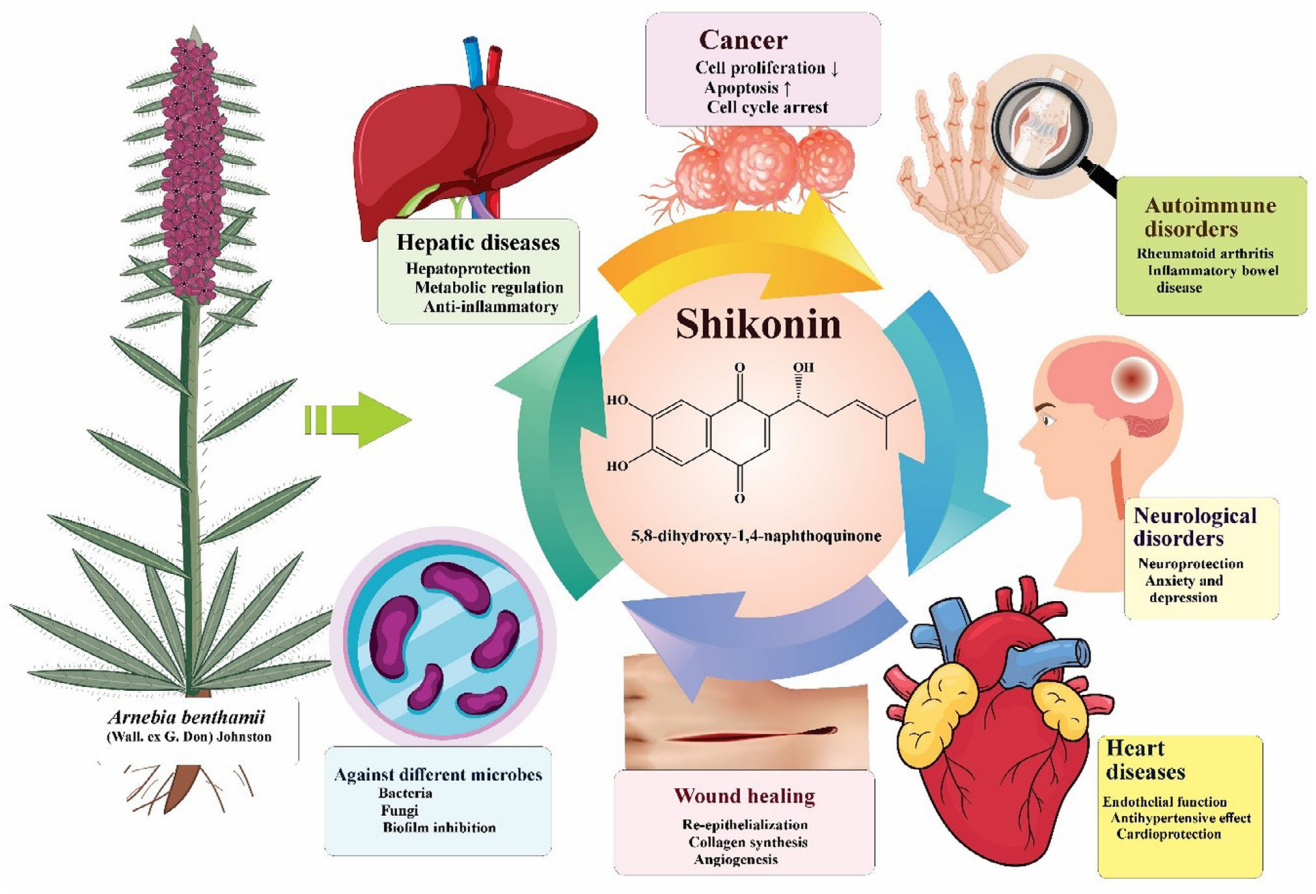


**Frontispiece**: SHK is depicted alongside its botanical source, molecular structure, and selected biological activities investigated in this review. The illustrated anticancer, anti‐inflammatory, antimicrobial, wound‐healing, neurological, cardiovascular, and hepatic effects reflect predominantly preclinical evidence and do not establish clinical efficacy.

## 2 Sources and Biosynthesis

The plant parts of *Arnebiab enthamii*, the extraction approaches used, and its reported biological activities are summarised in Figure 1.

**FIGURE 1:**
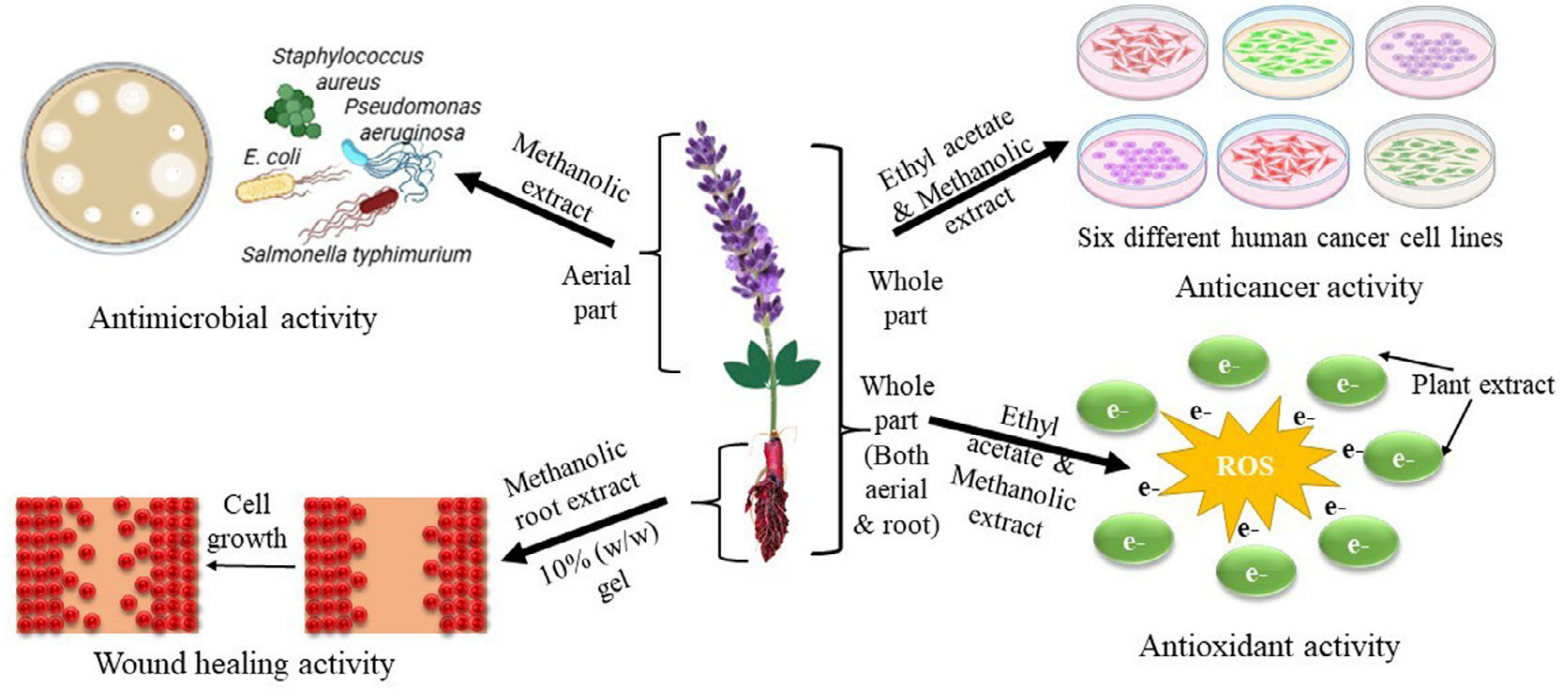
Schematic representation of the plant parts of *Arnebiab enthamii* (Wall. ex. G. Don) Johnston, the extraction approaches used, and the reported antimicrobial, anticancer, antioxidant, and wound‐healing activities.

### 2.1 Botanical Sources and Chemical Occurrence

The natural occurrence of SHK and its acylated derivatives is concentrated in Boraginaceae, particularly in pigmented root tissues. Reported producer genera include *Alkanna*, *Arnebia*, *Echium*, *Lithospermum*, and *Onosma* [17]. Frequently investigated species include *A. tinctoria*, *Arnebia benthamii*, *A. euchroma*, and *L. erythrorhizon*. These plants contain SHK together with derivatives such as acetylshikonin, deoxyshikonin, isobutyrylshikonin, isovalerylshikonin, and β,β‐dimethylacrylshikonin [2, 18].

The composition and reported yield vary with species, provenance, tissue, developmental stage, extraction method, and analytical procedure (Table 1). Comparative HPLC analysis of Boraginaceae herbs quantified multiple naphthoquinone derivatives across nine species and illustrates why yields cannot be generalised across sources [3]. This chemical variation is biologically relevant because cellular pharmacology studies of SHK derivatives report differences in biological response [4]. Cell‐cultured roots of *L. erythrorhizon* can also accumulate substantial amounts of these pigments [33]. Comparisons require authenticated material, matched tissue and extraction conditions, and common quantitative standards.

**TABLE 1:** Botanical sources within Boraginaceae, extraction approaches, reported constituents, quantitative estimates, and geographical origins.

S. no.	Botanical source	Extract formed	Compounds reported	Amount reported	Geographical location	References
1	*Alkanna tinctoria* (L.) Tausch	Ethanolic extract	Alkannin, acetylalkannin, angelylalkannin, 5‐methoxyangelylalkannin, dimethylacrylalkannin, arnebifuranone, alkanfuranol, and alkandiol	—	Budapest, Hungary	[19]
2	*Arnebia decumbens* L.	Hexane extract of the root	SHK	—	Iraqi dessert	[20]
3	*Arnebia euchroma* (Royle) Johnst., *A. guttata* Bunge, and *Lithospermum erythrorhizon* Sieb et Zucc.	Methanolic extract of the root	SHK, acetylshikonin (AS), deoxyshikonin (DS), β‐acetoxyisovalerylshikonin (ATS), isobutylshikonin (IBS), β,β‐dimethylacrylshikonin (DAS), 2‐methyl‐n‐butyrylshikonin (MBS), and isovalerylshikonin (IVS)	SHK (0.86%), AS (0.64%), DS (0.97%), ATS (0.65%), IBS (0.45%), DAS (0.34%), MBS (0.44%) and IVS (0.63%)	Mainland China	[3]
4	*A. euchroma* (Royle)	Ethanolic extract of dried root	Arnebiabinone; ethyl 9‐(20,50‐dihydroxyphenyl) nonanoate; octyl ferulate	—	Urumqi, Xinjiang Province of China	[21]
5	*A. euchroma* (Royle)	Ethanolic extract of the root	Meroterpenoids 2, 5, 7, 8, and 9	—	Haozhou City, Anhui Province, China,	[22]
6	*A. euchroma* (Royle)	—	Arnequinol A and arnequinone A, two unique meroterpenoids	—	Beijing, China	[23]
7	*Arnebia hispidissima* (*Lehm.*) *DC.*	Petroleum extract of dried root	Polycyclic geranyl hydroquinone‐derived metabolites such asarnebacene, and arnebidin; arnebin‐7 and vanillic acid (phenolic metabolites)	—	Wady Khashaba, South of Sinai Peninsula, Egypt	[24]
8	*A. hispidissima* (Lehm.) DC	Hexane extract of the root	Naphthoquinones: alkannin, arnebin‐1, arnebin‐7, arnebinol, cycloarnebin‐7, and tiglic acid (ester of dihydroxy alkannin)	Arnebin in vivo (0.62%); in vitro (0.038%, 0.069%, 0.086% and 0.078% in 2, 4, 6, and 8 weeks old callus, respectively)	Agricultural Research Station, Durgapura, Jaipur	[25]
9	*Lithospermum canescens* (Michx.) Lehm.	Hairy root	Acetylshikonin (ACS), isobutyrylshikonin (IBS), isovalerylshikonin, α‐methylbutyryl‐shikonin, pyrrolizidine alkaloids	Approximately 10% of ACS and IBS were reported in hairy roots than in natural roots of *L. canescens*	Poland	[26]
10	*L. erythrorhizon*	Ethanolic extract	Acetylshikonin, β‐sitosterol, β,β‐dimethylacrylshikonin, deoxyshikonin, and SHK	—	Shangai, China	[27]
11	*L. erythrorhizon*	Extract using various solvents (acetone, chloroform, ether, IPA, hexane and toluene	SHK	2%	Osaka, Japan	[28]
12	*L. erythrorhizon*	Ethanolic extract of the root	N1″, N3″‐dicoumaroylspermidine, erythrin A and erythrin B (novel compound), 19 other well‐known compounds (SHK, etc.)	—	Kyungdong Seoul, Korea	[29]
13	*Onosma leptantha*	Cyclohexane extract of air‐dried root	β,β‐dimethylacrylshikonin, isovalerylshikonin and acetylshikonin	—	Mount Taygetos Greece	[30]
14	*Onosma lycaonica* Hub. ‐Mor. *and O. papillosa* Riedl	Methanolic extract of the root	A total of 15 compounds were found, of which rosmarinic acid in *O. lycaonica* and hesperidin in *O. papillosa* were present in the highest concentrations.	*O. lycaonica* (3.52) *O. papillosa* (4.02)	Sertavul Pass, Mut, Mersin‐Turkey	[31]
15	*Onosma bracteatum* Wall.	Methanolic extract of the whole plant	1,7‐dihydroxy‐3‐methyl‐9*H*‐xanthene‐9‐one	—	Vyans Valley, Pithoragarh, Uttarakhand, India	[32]

### 2.2 Biosynthetic Pathway

SHK biosynthesis joins an aromatic precursor, *p*‐hydroxybenzoic acid (PHB), with the isoprenoid precursor geranyl pyrophosphate (GPP). PHB derives from phenylalanine through phenylpropanoid‐related metabolism, whereas GPP is supplied by isoprenoid metabolism through the mevalonate and/or nonmevalonate routes [2, 18, 34]. This convergence explains why SHK production is sensitive to both aromatic‐amino‐acid metabolism and isoprenoid flux.

Manipulation of mevalonate‐pathway flux can influence SHK production in cell cultures [35]. The membrane‐bound 4‐hydroxybenzoate 3‐geranyltransferase joins *p*‐hydroxybenzoate and geranyl pyrophosphate to form an early committed intermediate [34, 36]. Subsequent transformations involve oxidation and cyclisation; available reviews identify candidate cytochrome P450 reactions but do not establish every downstream enzyme or the complete sequence [2, 34].

The purification of a membrane‐bound prenyltransferase and experiments expressing a bacterial ubiA gene support the importance of membrane‐localised prenylation and precursor supply [36, 37]. Other downstream oxidation, cyclisation, transport, and acylation steps remain incompletely resolved [38]. Figure 2 should therefore be read as a working model rather than a fully established enzymatic sequence; recent characterisation of SHK/alkannin acyltransferases refines, but does not close, these gaps [17].

**FIGURE 2:**
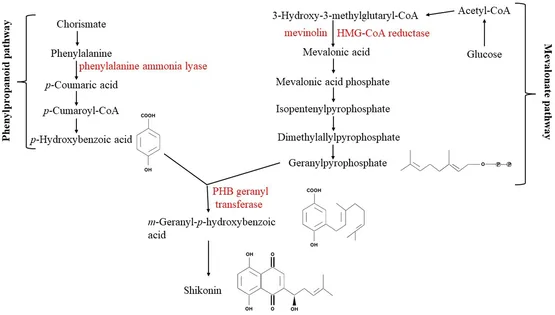
Proposed biosynthetic route to SHK from *p*‐hydroxybenzoic acid and geranyl pyrophosphate, highlighting the convergence of phenylpropanoid‐related and isoprenoid pathways [18, 33, 38].

## 3 Pharmacological Activities of SHK

SHK has been investigated for anticancer, anti‐inflammatory, wound‐healing, neuroprotective, antimicrobial, cardioprotective, hepatoprotective, and antioxidant effects. These activities are summarised in Figure 3 and discussed below. Because many of the same redox‐sensitive and inflammatory pathways recur across disease models, the evidence is organised by therapeutic context while emphasising shared mechanisms and indication‐specific limitations.

**FIGURE 3:**
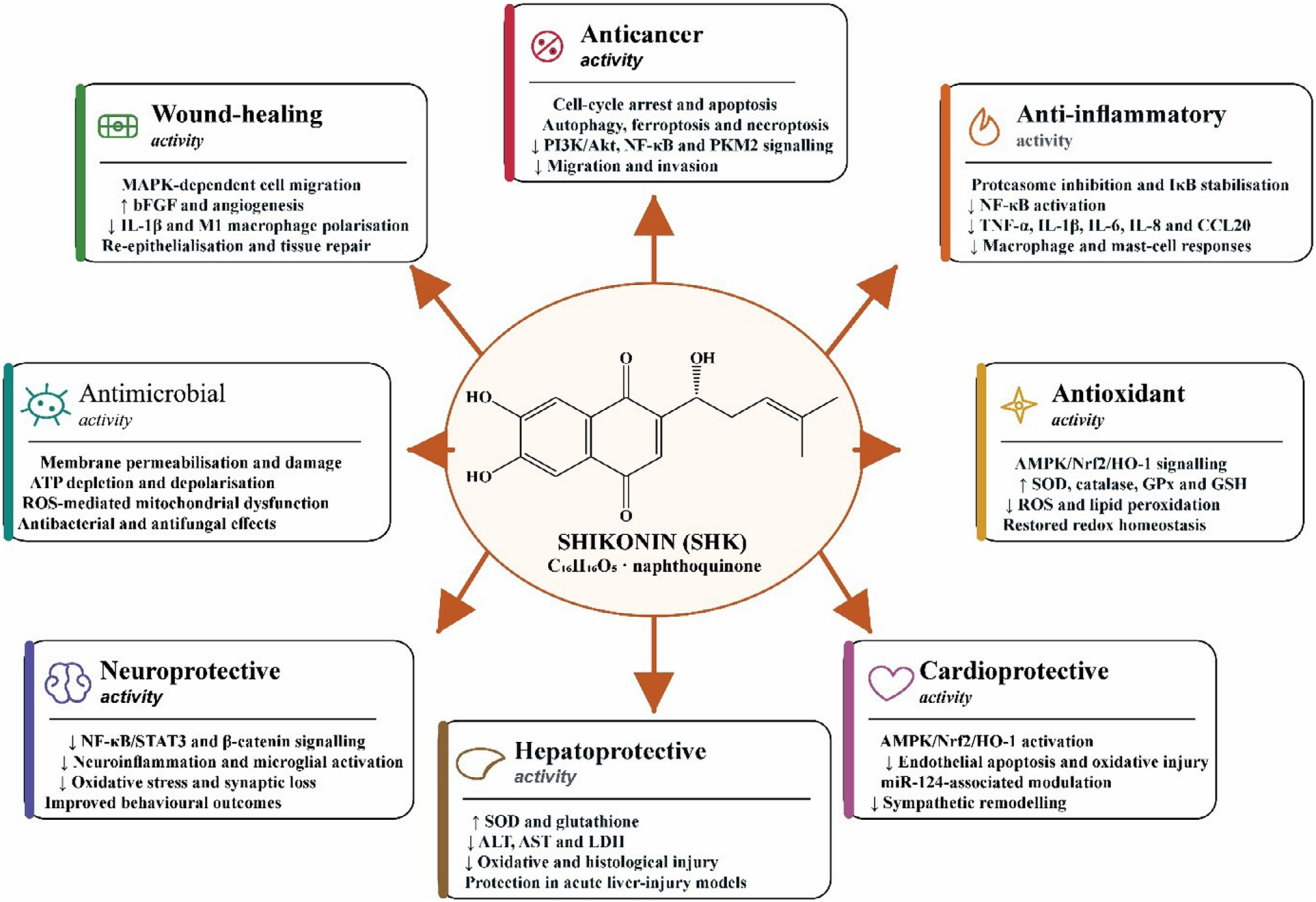
Overview of the pharmacological activities of SHK discussed in this review, including anticancer, anti‐inflammatory, wound‐healing, neuroprotective, antimicrobial, cardioprotective, hepatoprotective, and antioxidant effects, with representative molecular mechanisms.

### 3.1 Anticancer Activity

Cancer is the most extensively studied therapeutic context for SHK. Across tumour models, its reported effects converge on redox imbalance, suppression of proliferative signalling, cell‐cycle disruption, apoptosis, autophagy, ferroptosis, necroptosis, inhibition of migration and invasion, and, in selected formulations, immunogenic cell death. Frequently implicated networks include PKM2/AMPK, HIF‐1, PI3K/Akt, MAPK/JNK, NF‐κB, and RIPK1/RIPK3/MLKL [39, 40, 41]. However, most reports use a single‐cell line or xenograft and differ in exposure time, assay format, and formulation; mechanistic convergence is therefore stronger than evidence for efficacy in any specific human cancer.

#### 3.1.1 Mechanisms of Anticancer Action

##### 3.1.1.1 Inhibition of Cell Proliferation

Pure SHK generally inhibits tumour‐cell proliferation in the low‐micromolar range, although potency varies markedly among models. Reported IC_50_ values include 4.20–11.50 µM in thyroid cancer cells, 9.63 ± 0.43 and 13.35 ± 0.57 µM in two gastric cancer cell lines, and 1.40–2.20 µM across MDA‐MB‐231, U2OS, A549, and PANC‐1 cells [42, 43, 44]. By contrast, the IC_50_ value of 2.73 µg/mL reported for a hexane root extract of *L. erythrorhizon* cannot be directly compared with values for purified SHK because the extract contains multiple constituents [45]. Dose‐ and time‐dependent inhibition is reproducible across models, but cross‐study ranking is confounded by cell lineage, exposure duration, assay chemistry, compound purity, and serum conditions. Potency claims should therefore be based on molar concentrations of chemically characterised SHK and paired with nonmalignant controls.

##### 3.1.1.2 Induction of Apoptosis

Apoptosis is a regulated cell‐death programme executed through mitochondrial and/or death‐receptor pathways, with caspase activation serving as a common downstream event. Across gastric, colorectal, hepatic, and primary‐effusion‐lymphoma models, SHK commonly increased ROS, reduced mitochondrial membrane potential, promoted release or nuclear translocation of mitochondrial death factors, and activated caspase‐3 and caspase‐9 [7, 43, 46, 47]. In colorectal cancer cells, SHK also activated PERK/eIF2α/ATF4/CHOP and IRE1α/JNK signalling, downregulated the antiapoptotic protein BCL‐2, and increased caspase‐3/9 activation and PARP cleavage [48]. The attenuation of cytotoxicity by ROS scavenging in several models places redox disruption upstream of mitochondrial and endoplasmic‐reticulum stress; nevertheless, genetic or pharmacological rescue experiments are required to distinguish causal pathways from secondary stress responses.

##### 3.1.1.3 Cell‐Cycle Arrest

Cell‐cycle arrest is a checkpoint‐mediated pause at the G1/S, S, or G2/M transition that prevents DNA replication or chromosome segregation when mitogenic conditions or genome integrity are inadequate. Progression is coordinated by cyclin‐dependent kinases (CDKs) and their cyclin partners, whereas Cdc25 phosphatases activate CDK1 and are critical for entry into mitosis [49]. In HCT116 colon cancer cells, SHK suppressed HIF‐1 signalling and reduced proliferation [50]. In other cancer models, SHK inhibited Cdc25 activity, maintained CDK1 in an inhibitory hyperphosphorylated state, and increased accumulation in G2/M [44, 51]. Together, these findings support interference with checkpoint control, but the phase of arrest varies with cell type, dose, and exposure time. Future work should distinguish reversible arrest from senescence and irreversible commitment to cell death by using washout experiments and phase‐specific molecular markers.

##### 3.1.1.4 Inhibition of Cell Migration and Invasion

Migration describes cell movement, whereas invasion additionally requires penetration of extracellular‐matrix barriers. SHK reduced migration and invasion in glioblastoma cells by suppressing matrix‐metalloproteinase‐associated signalling and phosphorylation of β‐catenin and PI3K/Akt [52]. In papillary thyroid cancer cells, inhibition of DNMT1 and restoration of PTEN signalling were associated with reduced motility, while downregulation of the glycolytic regulator PFKFB2 accompanied reduced migration and invasion in lung cancer cells [53, 54]. These studies point to convergent effects on adhesion, matrix remodelling, epigenetic regulation, and tumour metabolism; however, most rely on 2D migration assays. Validation in 3D matrices, organoids, and metastatic models is needed before an antimetastatic effect can be inferred.

##### 3.1.1.5 Autophagy, Necroptosis, and Cellular Antitumour Immunity

Autophagy is a lysosome‐dependent recycling process that may protect stressed tumour cells or contribute to cell death depending on context. Necroptosis is a regulated lytic death programme typically mediated by RIPK1/RIPK3‐dependent activation of MLKL. SHK has been associated with autophagy and necroptosis in nonsmall‐cell lung cancer [55], RIPK1/RIPK3/MLKL upregulation in nasopharyngeal carcinoma [56], and galectin‐1/JNK‐linked autophagy in colorectal cancer [57]. Here, cellular immunity refers to antigen‐presenting‐cell activation and recruitment of effector lymphocytes following immunogenic tumour‐cell death. In a 4T1 model, an MUC1‐targeted nanocomplex codelivering SHK and chitosan‐silver nanoparticles induced RIPK3/MLKL‐mediated necroptotic immunogenic cell death, promoted dendritic‐cell maturation, increased intratumoural CD8+ and CD4+ T cells, and reduced regulatory T cells [9]. A self‐delivering SHK/curcumin nanobooster likewise intensified endoplasmic‐reticulum stress, dendritic‐cell maturation, and cytotoxic‐T‐lymphocyte recruitment [58]. These immune effects were demonstrated with engineered combination systems and should not be generalised to free SHK without direct comparative evidence.

Taken together, the experimental findings summarised in Table 2 reveal overlapping but model‐dependent responses to SHK, encompassing caspase‐mediated apoptosis, oxidative and endoplasmic‐reticulum stress, CDK1‐associated cell‐cycle arrest, p38‐linked autophagy, and necroptosis‐related signalling. Although these convergent processes support the mechanistic versatility of SHK, differences in cancer models, experimental designs, and outcome measures limit direct comparisons and emphasise the need for exposure‐matched and mechanistically validated studies before translation.

**TABLE 2:** Experimental models, molecular mechanisms, and reported anticancer actions of SHK.

S.no.	Model/cancer cell lines	Mechanism	Pharmacological action	References
1	BGC‐823 and SGC‐7901 (gastric cancer cell line) (in vitro) Immuno‐deficient nude mice xenograft bearing SGC‐7901 (in vivo)	SHK activates the caspase cascade. Inhibitors of caspase 3 and 9 ↓ SHK induced apoptosis (SGC‐7901) SHK ↓ size and weight of xenograft, ↑ Malondialdehyde (MDA)	↑ Caspase‐dependent apoptosis ↑ Lipid peroxidation, ↑ apoptosis in vivo	[43]
2	HCT116 and SW480 (colon cancer cell line) (in vitro) Xenograft models bearing SW480 in immuno‐deficient mice (in vivo)	↑ Caspase 3 and caspase 9 activity, ↓ expression of Bcl‐2 and Bcl‐xL ↑ Malondialdehyde (MDA)	↑ Program cell death (apoptosis) of colon cancer cells ↑ Oxidative stress in animal models	[46]
3	HCCLM3 (hepatocellular carcinoma cell lines)	↓ MMP ↑ ROS production and H_2_O_2_, ↓ intracellular ATP level, ↓ expression of PKM2, ↑ p‐AMPK and PGC1 ↑ Expression of cleaved caspase‐3, ↓ Expression of Bcl‐2 and p53	↑ Apoptosis ↑ Oxidative stress Regulatory effect on mitochondria	[47]
4	HCT‐116 and HCT‐15 (colorectal cancer cell lines)	Tumour growth inhibition rates were 52.3% and 67.8%, respectively. ↑ Expression of ATF4, BiP, CHOP, and p‐JNK	↓ Tumour growth in vivo Induced endoplasmic reticulum stress	[48]
5	K562 (myelogenous leukemia cell line), MCF‐7 (breast cancer cell line) and HeLa (cervical cancer cell line)	↓ Dephosphorylation of CDK1, ↓ CDK1	Cell cycle arrest	[51]
6	A375 (human melanoma)	↑ p‐p38, LC3B‐II, and Beclin 1 ↑ ROS	Induces autophagy via the p38 pathway ROS, mediated ER stress	[59]
7	A549 nonsmall cell lung cancer	↑ RIP1 protein ↑ Expression of cleaved PARP	Cell death via the necroptosis pathway PARP, mediated cell death	[55]
8	Human A549 nonsmall cell lung cancer	Upregulation of the expressions of cell cycleand apoptotic signalling‐regulatory proteins, upregulation of p53	Apoptosis, necrosis, and premature senescence	[60]

### 3.2 Anti‐Inflammatory Effects

Inflammation is a coordinated response to infection, sterile tissue injury, and altered cellular homeostasis. Across experimental models, SHK repeatedly converges on proteasome function, IκB stability, NF‐κB activation, and downstream cytokine production. In macrophages, proteasome inhibition contributed to the anti‐inflammatory action of SHK [8]. In lipopolysaccharide‐induced acute lung injury and mastitis models, SHK reduced IκB degradation and NF‐κB activation and lowered mediators, including TNF‐α, IL‐1β, and IL‐6 [61, 62]. Suppression of IL‐6, IL‐8, and CCL20 was also reported in human periodontal‐ligament cells [63]. The consistency of NF‐κB‐related effects supports a plausible anti‐inflammatory mechanism, but the relevant concentration, route, and selectivity differ among models; evidence for chronic autoimmune disease remains preclinical and should not be presented as established clinical efficacy.

### 3.3 Wound Healing

Wound repair requires coordinated haemostasis, inflammation, cell migration, angiogenesis, re‐epithelialisation, matrix deposition, and remodelling [64]. SHK enhanced repair in experimental skin wounds while reducing IL‐1β and increasing basic fibroblast growth factor, and it accelerated diabetic wound healing in rats by shifting macrophage polarisation away from an M1‐dominant phenotype and modulating MAPK signalling [11, 65]. These findings link the anti‐inflammatory and regenerative actions of SHK, but formulation is a major determinant of local exposure. Future studies should separate direct effects on fibroblasts, keratinocytes, angiogenesis, and macrophages from indirect benefits arising from antimicrobial activity or the dressing material itself.

### 3.4 Neuroprotective Effects

Neuroprotective evidence for SHK is heterogeneous and remains confined to cellular and animal models. In neuropsychiatric systemic lupus erythematosus mice, SHK improved cognitive and histological outcomes and was associated with reduced β‐catenin signalling, neuroinflammation, microglial activation, and synaptic loss [66]. In an aluminium‐chloride model of Alzheimer‐like pathology, SHK improved behavioural and antioxidant endpoints and reduced oxidative and inflammatory markers [67]. In a mouse model of alcoholic encephalopathy, inhibition of NF‐κB/STAT3 was associated with increased neural progenitor activity and improved behavioural outcomes [68]. Although these observations implicate inflammatory and redox pathways, the disease models, dosing regimens, and endpoints are not directly comparable, and evidence of brain exposure, target engagement, and long‐term neurological safety is limited.

### 3.5 Antimicrobial Activity

SHK exhibits antibacterial and antifungal activity, with membrane disruption and energetic collapse emerging as recurrent mechanisms. SHK isolated from *L. erythrorhizon* inhibited methicillin‐susceptible and methicillin‐resistant *S. aureus*, with minimum inhibitory concentrations of 7.8–31.2 µg/mL, and subinhibitory effects were potentiated by membrane‐permeabilising agents or ATPase inhibitors [10]. Against foodborne *S. aureus*, minimum inhibitory and bactericidal concentrations were reported at 35–70 µg/mL; treatment depleted intracellular ATP, depolarised and damaged the membrane, and altered cell morphology [69]. Antifungal activity against *Candida albicans* has also been linked to ROS accumulation, mitochondrial depolarisation, apoptosis, and necrosis [70]. These results establish mechanistic plausibility, but clinical relevance requires pharmacodynamic targets, infection‐model efficacy, host‐cell selectivity, and resistance‐selection studies.

### 3.6 Cardioprotective Effects

Cardioprotective evidence is limited to a small number of mechanistically distinct preclinical models. In mice with transverse‐aortic‐constriction‐induced heart failure, SHK reduced sympathetic remodelling through a mechanism associated with miR‐124 [71]. In oxidised‐low‐density‐lipoprotein‐injured endothelial cells, SHK reduced apoptosis and oxidative injury through AMPK/Nrf2/HO‐1 signalling [72]. These findings suggest potential effects on neurocardiac remodelling and endothelial redox homeostasis, but they do not yet establish protection across cardiovascular diseases. Confirmation in models with measured systemic exposure, clinically relevant comparators, cardiac function, vascular outcomes, and long‐term safety is required.

### 3.7 Hepatoprotective Activity

In an acetaminophen‐induced acute liver‐injury model, pretreatment with SHK restored superoxide dismutase and glutathione, limited increases in serum alanine aminotransferase, aspartate aminotransferase, and lactate dehydrogenase, and reduced histological injury [73]. This single mechanistic context supports a potential hepatoprotective effect related to oxidative‐stress control; it does not demonstrate efficacy in chronic liver disease or treatment after established overdose. Postinjury dosing, interaction with standard *N*‐acetylcysteine therapy, exposure–response relationships, and direct hepatic toxicity should be evaluated before therapeutic claims are broadened.

## 4 Preclinical Evidence

### 4.1 Summary of In Vitro and In Vivo Evidence

Table 3 summarises representative in vitro and in vivo studies, but these evidence levels should not be weighted equally. Cell‐based experiments identify molecular plausibility and concentration–response relationships, whereas animal models add absorption, distribution, metabolism, tissue context, and tolerability. Even so, most SHK studies use exploratory designs, heterogeneous formulations, and disease‐specific endpoints without pharmacokinetic matching. The principal translational question is therefore not whether SHK can produce a biological effect but whether a defined formulation can achieve an effective exposure in diseased tissue while maintaining an acceptable margin over normal‐tissue toxicity.

**TABLE 3:** Representative in vitro and in vivo studies of SHK across disease models, doses, and reported outcomes.

S.no.	Study type	Disease	Disease model	Dosage	Outcome	References
1	In vitro	Obesity	3T3‐L1 mouse fibroblast cells	1.2 µM	Reduces adipogenesis by suppressing mir‐34a, a mRNA expression of FKBP1B	[74]
2	In vitro	Fungal infection	*C. albicans* SC5314 and *C. albicans* SN250	4 μg/mL	Enhanced the accumulation of nitric oxide (NO) and decreased the transcription level of YHB1 and CTA4 function via NO‐mediated damage	[75]
3	In vivo	Gall bladder cancer	Nude mice	1.3 mg/kg	Promote apoptosis and G0/G1 phase arrest, induce apoptosis via the JNK pathway, elicit antitumour effect both in vivo and in vitro	[76]
4	In vitro	Gall bladder cancer	Human gallbladder cancer cell lines NOZ and EHGB‐1	0.5–4 μM		
5	In vivo	Bacterial pneumonia	*S. pneumoniae* D39	0.5–8 μg/mL	Decreased Pneumolysin (PLY)‐induced haemolytic activity, genotoxicity, and cytotoxicity; Reduced the mice mortality due to lethal pneumonia and attenuated the injury of target organs	[77]
6	In vivo	Hypertrophic scars	Mini pigs	10 μg (sprayed onto surface)	Reduces the thickness of the epidermis and dermis layer, reduced keratinocytes, inhibits p63 expression, cytokeratin 10, α‐smooth muscle actin, TGF‐β 1, and collagen I	[78]
7	In vitro	Ovarian cancer	Cisplatin‐sensitive and ‐resistant human ovarian carcinoma (A2780) cells	2.5–40 μM	Induced mitochondria‐mediated apoptosis and attenuation of epithelial‐mesenchymal transition	[79]
8	In vitro	Lung cancer	Nonsmall cell lung cancer cell lines A549 and H1299	2.4 and 3.6 μM	Antagonistic effect associated with PKM2/STAT3/cyclinD1 mechanisms enhanced for anti‐tumour response	[80]
9	In vitro	Hepatocellular carcinoma	Human hepatocellular carcinoma cell linesSMMC‐7721	1–4 μM	Induce apoptosis via stimulating intracellular calcium homeostasis, activation of caspases −3, −8, −9, and ROS and mitochondrial dysfunction	[81]
10	In vitro	Osteoporosis	Bone marrow‐derived monocyte	0.2–48.6 μM	Decrease in osteoclastogenesis by RANKL‐mediated signalling pathways, specifically reduced activation of NF‐κB and MAPK	[82]
11	In vitro	Pancreatic ductal adenocarcinoma	Human pancreatic ductal adenocarcinoma cell line (PaCa‐2)	0.5–5 μM	Inhibition of ATP depletion, PMCA, and cytotoxic Ca2+ overload reduced PDAC cell proliferation and migration and induced cell death	[83]
12	In vivo	Multiple sclerosis	C57BL/6 mice	20 mg/kg	Reduced the expression levels of Bax, IFN‐γ, and TNF‐α, while increased the expression levels of TGF‐β, Bcl2 and the activity of glutathione peroxidase‐1 (GPX‐1) enzyme	[84]
13	In vitro	Skin disease	Human burn skin	5–20 μM	Targeted NLRP3‐mediated inflammation, decreased level of cleaved caspase‐1 and mature IL‐1β and improved wound healing in vivo	[85]
14	In vivo	Skin disease	WT C57BL/6J and NLRP3‐KO (NLRP3^–/–^) male mice	10 mg/kg		
15	In vitro	Colorectal carcinoma	SW620 cells and HCT116 cells	3–12 μM	Promotes cell apoptosis and autophagy by interfering with the galectin‐1 and JNK signalling pathway	[57]
16	In vitro	Liver fibrosis	LX‐2 cells and Mouse hepatic stellate cell	0.01–1 μM	Inhibitory effect on HSC activation, reduction in aerobic glycolysis, and decrease in *MYC* and *CCND1* expression via regulating histone H3K9 acetylation, which attenuates liver fibrosis in vivo	[86]
17	In vitro	Breast cancer	Triple‐negative breast cancer (TNBC) cell MDA‐MB‐231 and 4T1	0.3, 1, and 3 μM	Inhibitory effect on IMPDH2, resulting in reduced cell proliferation in TNBC	[87]
18	In vitro	—	*Streptococcus mutans* strain MT8148 biofilm	20 and 200 μM	Inhibitory effect on *S. mutans* activity (growth and biofilm formation) and nonmutans Streptococci growth	[88]

#### 4.1.1 In Vitro Evidence

The in vitro literature is dominated by cancer cell lines, in which SHK affects mitochondrial function, ROS production, cell‐cycle control, apoptosis, ferroptosis, proteasome activity, migration, and invasion [48, 51, 89, 90]. Antifungal studies, particularly in *C. albicans*, extend the evidence beyond oncology [70]. These experiments are useful for hypothesis generation, but they frequently lack matched nonmalignant cells, measurement of unbound drug, orthogonal viability assays, genetic validation of proposed targets, and clinically relevant exposure durations. Repeating lists of responsive cell lines therefore adds less value than identifying mechanisms that remain robust across models and conditions.

#### 4.1.2 In Vivo Evidence

In vivo studies provide evidence of antitumour, immunomodulatory, and wound‐healing activity but remain heterogeneous. SHK reduced tumour growth and altered splenic CD^8^^+^ and regulatory‐T‐cell populations in a 4T1 breast‐cancer model, while xenograft studies in other cancers linked tumour suppression to apoptosis or ferroptosis [89, 90, 91]. Local delivery systems have also been explored to improve wound exposure and retention. A 2025 preprint reported a SHK‐loaded cyclodextrin‐nanosponge/chitosan–poly(vinyl alcohol) hydrogel with sustained release and wound‐healing effects; because this evidence is not yet a peer‐reviewed journal article, it should be identified explicitly as a preprint and updated if a final publication becomes available [92]. Across models, formulation, administration route, dosing schedule, and tumour or wound characteristics differ substantially, and few studies relate tissue exposure to efficacy and systemic toxicity.

### 4.2 Clinical Evidence and Prospects for Human Application

Human evidence remains extremely limited. A frequently cited 1991 report described 19 patients with advanced lung cancer who received a SHK‐containing combination and reported an overall response rate of 63.3%, a remission rate of 36.9%, and 1‐year survival of 47.3% [12]. However, the small sample, combination treatment, absence of a contemporary randomised comparator, limited methodological reporting, and lack of modern trial registration prevent attribution of the outcomes to SHK. This report should therefore be treated as historical, hypothesis‐generating evidence rather than proof of clinical efficacy. Commercial or traditional topical use likewise cannot substitute for trials of a chemically defined product with standardised dose, purity, exposure, and safety monitoring.

The major translational barriers are poor aqueous solubility, chemical and photochemical instability, rapid metabolism, uncertain oral absorption, route‐dependent toxicity, and nonselective distribution [1, 93]. Liposomes, polymeric micelles, nanoparticles, nanogels, nanoemulsions, and related carriers can improve apparent solubility, protect SHK during circulation, and enable targeted or stimulus‐responsive release. However, clinical readiness also requires human‐compatible excipients, reproducible manufacturing, stability during storage, validated pharmacokinetics, and evidence that the carrier improves therapeutic index rather than merely increasing total drug accumulation.

Current evidence does not support describing purified SHK as an established systemic therapy. Topical products containing botanical extracts or combination ingredients do not define the exposure, purity, or contribution of SHK and cannot be extrapolated to oral or parenteral use. Before first‐in‐human development, priority studies should establish metabolite profiles, absolute bioavailability, protein binding, organ distribution, genotoxicity, reproductive and repeated‐dose toxicity, and formulation‐specific safety. Early clinical trials should then use exposure‐guided dose escalation, pharmacodynamic biomarkers, and indication‐specific endpoints.

## 5 Toxicity and Safety Concerns

The safety profile of SHK is difficult to define because studies differ in compound identity, purity, derivative, formulation, route, exposure duration, and toxicological endpoint. In normal V79 cells, Figat reported SHK EC_50_ values of 0.18–0.60 mg/L across LDH, MTT, and neutral‐red‐uptake assays, whereas acetylshikonin values were 0.49–1.32 mg/L [13]. The assay‐dependent range itself illustrates that a single viability endpoint is insufficient. Reports of limited oral toxicity and greater toxicity after intraperitoneal or intravenous administration are plausibly related to route‐dependent absorption and peak systemic exposure, but they should not be interpreted as evidence that oral SHK is inherently safe [2].

### 5.1 Cytotoxicity and Dose‐Dependent Effects

The general statement that SHK is therapeutic at low doses but toxic at high doses is too broad without specifying exposure, route, formulation, and endpoint. Su et al. reported negligible haematological and nonhaematological toxicity after repeated oral administration of SHK derivatives at doses up to 800 mg/kg/day for 90 or 180 days [94]. This negative result does not support a conclusion of high‐dose toxicity; rather, it indicates that toxicity is conditional on compound identity, formulation, route, bioavailability, duration, and the sensitivity of the measured endpoints. It should be interpreted alongside normal‐cell cytotoxicity and parenteral‐exposure studies. Future dose–response assessments should therefore quantify systemic exposure and organ‐specific injury instead of comparing nominally administered doses alone.

Cell‐based concentration–response data further show why efficacy and safety must be assessed together. In HepJ5 and Mahlavu hepatocellular‐carcinoma cells, SHK produced IC_50_ values of ≈2 µM, while lower exposures reduced migration before extensive loss of viability [95]. In nonmalignant HaCaT keratinocytes, the reported IC_50_ decreased from 6.34 µM at 24 h to 2.43 µM at 48 h, demonstrating both concentration‐ and time‐dependent toxicity [96]. Because the studies used different cell types and protocols, these values cannot define a therapeutic window. Direct comparisons require tumour and matched normal cells tested under the same exposure conditions with orthogonal viability and mechanism‐based assays.

Mechanistically, SHK cytotoxicity is consistent with excessive ROS generation, mitochondrial depolarisation, endoplasmic‐reticulum stress, BCL‐2 downregulation, PARP cleavage, and activation of caspase‐3/9 [48]. These processes can be therapeutically useful in tumour cells but are not tumour‐specific. The same redox and membrane‐reactive properties may injure normal tissues when local or systemic exposure is high. A clinically meaningful safety assessment must therefore combine target engagement in diseased tissue with biomarkers of oxidative injury, mitochondrial dysfunction, inflammation, and organ toxicity in normal tissues.

Reported tumour‐cell potency also should not be conflated with systemic toxicity. SHK produced an IC_50_ value of ≈3 µM after 48 h in SNU‐407 colon‐cancer cells and suppressed proliferation in A‐498 and CAKI‐2 renal‐carcinoma cells at 2.5 µM [97, 98]. Acetylshikonin caused G2/M arrest and reduced viability in chondrosarcoma cells with an IC_50_ value of 1.3 µM [99]. These results demonstrate anticancer potency, not an acceptable therapeutic index. Matched normal‐cell comparisons, free‐versus‐formulated exposure, clonogenic recovery, and in vivo pharmacokinetic/toxicodynamic modelling are needed to identify a dose range that is both effective and tolerable.

### 5.2 Safety Evaluation in Animal Models and Clinical Settings

Formal animal‐safety studies of chemically defined SHK remain scarce. Many publications were designed to demonstrate efficacy and report only gross tolerability, body weight, or limited histology; such observations are not equivalent to repeated‐dose toxicology. The long‐term oral study of SHK derivatives by Su is reassuring for the tested material and route, but it cannot resolve the safety of purified SHK, parenteral formulations, or nanocarrier‐associated changes in distribution [94]. A translational programme should include toxicokinetics, complete clinical chemistry and haematology, organ histopathology, safety pharmacology, genotoxicity, immunotoxicity, and recovery groups under a standardised formulation.

Efficacy‐oriented animal studies should also be interpreted separately from safety studies. Intragastric SHK reduced inflammation in mice, topical or local treatment promoted burn repair, and SHK suppressed tumour growth in a small‐cell lung cancer xenograft [100, 101, 102]. These outcomes support pharmacological activity but do not establish systemic safety unless adverse clinical signs, organ function, histopathology, exposure, and delayed toxicity are assessed prospectively. The absence of reported adverse events is not evidence of the absence of toxicity.

Disease‐specific models provide additional signals but remain insufficient for broad safety conclusions. SHK reduced rotenone‐associated injury in SH‐SY5Y cells, and antibiofilm studies in skin‐infection models combined microbial inhibition with tissue regeneration [103, 104]. The former requires confirmation in vivo with brain exposure and neurological safety, whereas the latter mainly supports local application. Future investigations should distinguish protective pharmacology from direct toxicity, carrier‐related effects, and changes in metabolism and should evaluate both acute and delayed outcomes.

Overall, the available literature supports continued preclinical development but not a definitive human safety profile. Evidence from female‐cancer models, wound‐healing formulations, and branded botanical products cannot be extrapolated across indications or routes [105, 106]. Purified SHK, botanical extracts, derivatives, and nanoformulations should be treated as distinct products. Each requires independent characterisation of composition, pharmacokinetics, benefit–risk balance, and manufacturing consistency before clinical claims are made.

## 6 Drug Formulations and Delivery Approaches

Formulation is an enabling component of SHK development because the free compound combines low aqueous solubility and chemical instability with nonselective distribution. Investigated approaches include liposomes, polymeric micelles, polymeric and metal‐coordination nanoparticles, lipid carriers, cyclodextrin complexes, hydrogels, and combination or stimulus‐responsive systems. Their value should be judged by improvement in exposure, selectivity, efficacy, safety, and manufacturability rather than particle size or encapsulation efficiency alone.

### 6.1 Nanoformulations and Drug‐Delivery Strategies

Nanodelivery systems seek to increase apparent solubility, protect SHK from degradation, prolong circulation, alter tissue distribution, and control intracellular release. A comprehensive review of SHK nanomedicines describes liposomes, polymeric micelles, nanoparticles, nanogels, and nanoemulsions for long circulation, tumour targeting, microenvironment‐responsive release, and combination therapy [93]. These advantages remain formulation‐specific: increased total tumour accumulation does not by itself demonstrate a higher therapeutic index, and studies should compare free and encapsulated SHK at matched systemic and intratumoural exposure.

Representative systems illustrate different design objectives. Low‐dose SHK and anthracycline coloaded liposomes enhanced immunogenic cell death, whereas NGR‐modified PEG‐PLGA micelles were designed to target dendritic cells in allergic rhinitis [16, 107]. TEM1‐targeted PEGylated PLGA nanoparticles reduced toxicity toward T lymphocytes relative to free SHK, and hyaluronic‐acid‐coated SHK liposomes improved retention and antitumour effects in triple‐negative breast‐cancer models [108, 109]. Metal coordination and hybrid systems have additionally been used to improve dispersion, redox modulation, or tumour‐microenvironment responsiveness [110]. Across these studies, the carrier, cargo ratio, release kinetics, model, and comparator differ, preventing a reliable ranking of platforms.

Recent literature extends this design space. Self‐assembled SHK/curcumin nanoparticles amplified endoplasmic‐reticulum stress and immunogenic cell death in a colorectal‐tumour model [58]. A 2026 review of biomimetic cell‐membrane‐mediated delivery of natural products discusses immune evasion, homotypic targeting, and combination therapy as broader design principles [111]. A review of nanomedicine‐mediated remodelling of the colorectal tumour immune microenvironment provides a complementary translational framework [112]. Pullulan‐stabilised gliadin nanocomplexes loaded with astragaloside IV illustrate a protein–polysaccharide strategy for oral delivery of a poorly soluble natural product [113]. The latter three publications are relevant as platform or design precedents, not as direct evidence of SHK efficacy, and should be cited with that distinction made explicit.

Across formulations, commonly reported endpoints include particle size, encapsulation efficiency, in vitro release, cellular uptake, and short‐term efficacy. Clinical translation requires a more demanding comparative package: stability in biologically relevant media and during storage, unbound pharmacokinetics, biodistribution and clearance, carrier‐only controls, repeated‐dose organ toxicity, immunogenicity, batch reproducibility, sterilisation compatibility, scale‐up, and head‐to‐head comparison with free SHK and standard therapy. No SHK delivery platform has yet demonstrated this complete evidence chain.

As outlined in Table 4, SHK delivery platforms span different therapeutic objectives, from anticancer codelivery and redox‐responsive release to anti‐inflammatory, dermatological, wound‐healing, and dental antimicrobial applications. This diversity highlights the need to evaluate each formulation against indication‐specific efficacy and safety endpoints rather than assuming that improved encapsulation, local retention, or stimulus responsiveness necessarily confers a translational advantage.

**TABLE 4:** SHK formulations, intended therapeutic applications, proposed mechanisms, and supporting references.

S. no.	Drug formulations	Potential therapeutic application	Mechanism of action	References
1	Hybrid micelles codelivering SHK and IDO‐1 siRNA	Immunotherapy	Triggers immunogenic cell death and moderates the IDO‐1‐triggered immunosuppression	[114]
2	ROS‐activable prodrug nanoamplifier (CuSK@DTC‐PPB)	Antitumour/anticancer Therapy	Triggered release of SHK inside tumour cells, by chain activation of Cu binded DTC‐PPB. This, in turn, produces ROS and forms a positive feedback loop that targets only cancer cells, leaving healthy ones unharmed.	[115]
3	Nanostructure lipid carriers of alkannins and SHK	Dermal and cosmetic	Induce enhanced dermal cell viability and antioxidant cellular response by targeting its related genes	[116]
4	SHK‐loaded and hyaluronic acid (HA)‐modified hollow Fe‐MOF (HFM), HFM@SK@HA	Anticancer microwave therapy	HFM@SK@HA improves the heating effect at the tumour site and mediates the therapy's dynamics by catalysing endogenous H_2_O_2_ and ROS generation	[117]
5	Zn‐SHK‐PEG nanoparticles	Anti‐inflammatory treatment	Clears intracellular ROS through regulating Nrf2/HO‐1 pathway and inhibits NLRP3 inflammasome‐mediated inflammation and apoptosis activation by modulating AMPK/SIRT1 pathway during sepsis	[110]
6	PLA‐HPG‐based bio‐adhesive coating for SHK nanoparticles	Dermal bio‐adhesive	Prolonged SHK enhances fibroblast proliferation for treating wounds and synthesising collagen fibres	[118]
7	SHK‐loaded PLGA nanoparticles hydrogel	Dermal	Alleviated psoriasis and reduced concentration of inflammation inducing cytokines (IL17A, IL‐17F, IL‐22, IL‐1β, and TNF‐α)	[119]
8	SHK dispersion through encapsulated in β‐1,3‐1,6 Glucan	Odontic	Inhibits *S. mutans* and nonmutans *Streptococci* activity through dispersing SHK in toothpaste via in vitro analysis	[88]

### 6.2 Topical and Systemic Treatment Approaches

Topical administration is most closely aligned with the historical use of SHK‐containing botanicals and may limit systemic exposure. Zicao ointment promoted repair in a rat burn model, while electrospun polymer meshes, microcapsules, and cyclodextrin inclusion complexes have been used to improve local retention, stability, or aqueous dispersion [120, 121, 122, 123]. Nevertheless, traditional use does not establish a standardised SHK dose or contribution to efficacy. Topical products require dermal pharmacokinetics, irritation and sensitisation testing, microbiological quality, wound‐specific safety, and comparison with standard dressings. Systemic formulations face a higher evidentiary threshold, including absolute bioavailability, metabolite identification, organ distribution, repeated‐dose toxicity, and demonstration that targeting reduces rather than redistributes toxicity.

## 7 Conclusions and Future Directions

SHK is a mechanistically versatile naphthoquinone whose strongest evidence lies in preclinical modulation of redox homeostasis, regulated cell death, inflammation, microbial membranes, and tissue repair. The recurrence of ROS, mitochondrial and endoplasmic‐reticulum stress, NF‐κB/MAPK/PI3K‐Akt signalling, cell‐cycle control, and RIPK‐dependent necroptosis across models provides a coherent mechanistic framework. However, the literature remains fragmented by heterogeneous compound sources, formulations, exposure conditions, and endpoints. In vitro potency and xenograft efficacy should not be equated with clinical effectiveness, and evidence from a botanical extract, derivative, or nanocarrier cannot be attributed automatically to purified free SHK.

The next phase of development should prioritise chemically standardised material; validated stability‐indicating assays; route‐specific pharmacokinetics and metabolite profiling; matched efficacy and normal‐tissue toxicity; and mechanistic rescue experiments that distinguish primary targets from secondary stress responses. Delivery systems should advance only when they improve therapeutic index at matched exposure and can meet requirements for reproducibility, storage, sterilisation, scale‐up, and long‐term safety. Clinically relevant organoids, immunocompetent disease models, and exposure‐guided studies should precede well‐designed early‐phase trials. Until this evidence is available, SHK is best positioned as a promising therapeutic lead and formulation research platform, not an established medicine.

## Author Contributions

**Namini Joshi**: conceptualisation, literature search, data curation, writing – original draft, and manuscript compilation. **Prabhakar Semwal**: literature search, data curation, and writing – original draft. **Balwant Rawat**: data curation and literature validation. **Ravendra Kumar**: writing – review and editing. **Mozaniel Santana de Oliveira**: critical review, supervision, and manuscript editing. Nishant Rai: data curation and writing support. **Janhvi Mishra Rawat**: cnceptualisation, supervision, critical review, and final approval of the manuscript. All authors have read and agreed to the published version of the manuscript.

## Funding

This research was funded from University Grant Commission, Govt of India through Project number [82‐7/2022] (SA‐III).

## Conflicts of Interest

The authors declare no conflicts of interest.

## Data Availability

Data sharing is not applicable to this review because no new datasets were generated or analysed.
